# Correlation Analysis of C‐terminal telopeptide of collagen type II and Interleukin‐1β for Early Diagnosis of Knee Osteoarthritis

**DOI:** 10.1111/os.12586

**Published:** 2019-12-16

**Authors:** Cai‐xia Liu, Ge Gao, Xiao‐qun Qin, Chang‐qing Deng, Xiong‐jie Shen

**Affiliations:** ^1^ School of Integrated Chinese and Western Medicine Hunan University of Traditional Chinese Medicine Changsha Hunan China; ^2^ Faculty of Laboratory Medicine Third Xiangya Hospital, Central South University Changsha Hunan China; ^3^ Department of Physiology Xiangya School of Medicine, Central South University Changsha Hunan China; ^4^ Department of Spine Surgery Hunan Provincial People's Hospital Changsha Hunan China

**Keywords:** Correlation analysis, C‐terminal telopeptide of collagen type II, Interleukins‐1β, Kellgren‐Lawrence score, Knee osteoarthritis

## Abstract

**Objective:**

To analyze the correlation between the Kellgren–Lawrence (K‐L) score of knee osteoarthritis (KOA) patients with different degrees and their urine concentration of C‐terminal telopeptide of collagen type II (CTX‐II) and interleukin‐1β (IL‐1β), and to further evaluate the diagnostic value of CTX‐II and IL‐1β during the pathological process by producing an experimental osteoarthritis (OA) model in rabbits.

**Methods:**

From 1 January 2017 to 31 December 2018, a total of 34 subjects (7 mild, 9 moderate, 9 severe arthritis patients, and 9 healthy individuals) comprising 16 men and 18 women were included in this study. Patients were diagnosed according to the American College of Rheumatology (ACR) criteria. The urine of all subjects was collected to detect the concentration of CTX‐II and IL‐1β. The rabbits in the KOA group were subjected to protease (control group with saline) injection into the articular cavity of their right knees and immobilization with gypsum. We used radiological and histological examination to identify the KOA model. ELISA was applied to investigate the concentrations of CTX‐II and IL‐1β in urine and serum, and Spearman's rank correlation analysis was used to analyze the correlation.

**Results:**

There was no significant difference in the mean ages and body mass index (BMI) between groups. The mean ages of mild, moderate, and severe arthritis patients and healthy individuals were 54.29 ± 5.76, 58.44 ± 6.44, 59.89 ± 6.75, and 56.67 ± 4.18 years, respectively. The mean BMI of mild, moderate, and severe arthritis patients and healthy individuals were 23.59 ± 1.56, 23.57 ± 2.06, 24.46 ± 1.64, and 23.42 ± 1.35 kg/m^2^, respectively. The Kellgren–Lawrence (K‐L) score was higher with the aggravation of KOA. The K‐L scores of mild, moderate, and severe KOA patients were 1.14 ± 0.38, 2.56 ± 0.53, and 3.63 ± 0.52, respectively. The KOA symptoms of patients became more severe, with not only increased K‐L scores but also elevated concentrations of CTX‐II and IL‐1β. Moreover, there was a positive correlation between CTX‐II and IL‐1β of all subjects (*r* = 0.974, *P* < 0.001), between K‐L score and urine concentration of CTX‐II (*r* = 0.900, *P* < 0.001), and between K‐L score and IL‐1β (*r* = 0.813, *P* < 0.001) of all subjects. Both were significantly increased in KOA group rabbits at all time points after surgery. The serum concentration of CTX‐II and IL‐1β was elevated as early as in the 2nd week (3.69 and 4.25 times) and reached a peak (5.41 and 7.23 times) in the 4th week after surgery. Then, until 12 weeks after surgery, the CTX‐II and IL‐1β concentrations in the KOA group were slightly reduced and remained around 4.5 and 6.3 times that in the control group. Moreover, there was a positive correlation between the serum concentration of IL‐1β and CTX‐II (*r* = 0.967, *P* < 0.001).

**Conclusion:**

CTX‐II and IL‐1β, which were significantly increased during the process of KOA, can be used as biomolecular markers to provide guidelines for early diagnosis and treatment of KOA.

## Introduction

Osteoarthritis (OA), which is characterized by damage and progressive degeneration of cartilage, accompanied by pain, osteophyte formation, and loss and damage of other structures surrounding the joint leading to the disability of joint, is the most common type of bone and joint degenerative disease^1^. Approximately 9.29% of 60‐year‐olds in the US population are diagnosed with knee OA^2^. OA leads to long‐term disability in adults and is associated with various complications, such as cervical spondylosis, lumbar disease, vertebral spondylolisthesis, vertebral compression fractures, and necrosis of the femoral head. The etiology and pathogenesis of OA involve a variety of risk factors, such as age, gender, genetic predisposition, obesity, and joint injury. There is no effective cure, with the treatment options for patients including symptomatic treatment, pain relief, and, finally, joint replacement^3^, ^4^. Despite the multifactorial effects of OA, the abnormal metabolism and structure remodeling of articular cartilage and a series of pathological changes in the joint synovium, subchondral bone, joint capsule, and the muscles around joint are common pathological manifestations of OA. The destruction and degeneration of cartilage, which can lead to permanent damage and loss of cartilage, obviously reduce the quality of life of patients. Cartilage does not easily repair itself after injury or degeneration and it cannot be cured with the existing treatments^5^. Therefore, early diagnosis of OA is crucial for its therapy and prognosis.

Currently, the etiology of OA is unclear and its diagnosis mainly relies on classical radiography and pathological examination, which usually reveal OA in the middle and late stages. Besides the use of clinical risk factors and symptoms for predicting OA, biomolecular markers, as another marker of arthritis progression, have attracted increasing attention for early diagnosis and intervention^6^. Another attractive alternative is the measurement of biochemical markers in blood, urine, or synovial fluid samples. Biomarkers are used to reflect the dynamic and quantitative changes in joint remodeling and disease progress. Structural molecules or fragments derived from joint cartilage, joint synovium and bone, which are affected by OA and may be specific to one type of joint tissue or common to them all, are most likely the best candidate biomarkers in the progression of OA^6^, ^7^, ^8^. With the development of molecular biology, there are many kinds of joint biomarkers that have been studied and identified, such as biomarkers related to differentiation and matrix production, matrix destruction, inflammation, and proteases^6^, ^8^, ^9^, ^10^, ^11^. These molecules can represent tissue synthesis and may be measured in synovial fluid, blood, or urine. Moreover, as a valuable biochemical, its one important characteristic should be that it can detect the early stages of OA. Biomarkers related to collagen metabolism and inflammation have attracted our attention.

C‐terminal telopeptide of collagen type II (CTX‐II) and interleukin‐1β (IL‐1β) are involved in articular cartilage degeneration. CTX‐II, the most abundant protein in cartilage, is a product of joint tissues (especially the cartilage extracellular matrix) and can be used to preliminary judge the degree of joint degeneration in OA^9^, ^12^. IL‐1β, which is secreted from synovial cells and inflammatory cells in OA, induces the erosion and proteolysis of the cartilage extracellular matrix by stimulating the production of proteolytic enzymes^13^, ^14^. There are some published studies on the biomarkers of OA but few have simultaneously measured these two biomarkers (CTX‐II and IL‐1β) in the pathological process of OA. We hypothesized that CTX‐II and IL‐1β can be used as markers for the early diagnosis and treatment of knee osteoarthritis (KOA). Therefore, the aim of this study is to evaluate the role of CTX‐II and IL‐1β in the process of KOA by combining data from clinical and experimental studies. Thus, in our study, we respectively analyzed the correlation between K‐L score of KOA patients with different degrees and their urine concentration of CTX‐II and IL‐1β. For further investigation of the role of CTX‐II and IL‐1β in the process of KOA, we produced a KOA model in rabbits by injecting collagenase into the knee joint, which specifically hydrolyzes the 3‐D helical structure of natural collagen under physiological PH and temperature conditions without damaging other proteins and tissues. Then, we confirmed the KOA model by X‐ray and pathological examination. In addition, we analyzed the serum concentration of CTX‐II and IL‐1β and performed a longitudinal analysis to evaluate their diagnostic value during the pathological process.

## Materials and Methods

### *Patients*

This study was approved by the ethics committee of our hospital. According to the American College of Rheumatology (ACR) criteria, patients diagnosed with knee OA between 1 January 2017 and 31 December 2018 through evaluation of their X‐ray images in our hospital were considered for our study. A total of 34 subjects (7 mild, 9 moderate, 9 severe arthritis, and 9 healthy individuals) were included: 16 men and 18 women. A signed informed consent form was obtained from all subjects in this study. Urine of all objects (patients and volunteers) was collected to detect the concentration of CTX‐II and IL‐1β.

### *Experimental Animals and Construction of Knee Osteoarthritis Model*

Ninety healthy mature New Zealand white rabbits (2.5–3.0 kg, male, 6 months old) were obtained from the Animal Experimental Center at Xiangya School of Medicine, Central South University. The rabbits were divided into two groups using a random number decimation method: a control group and a KOA group (45 rabbits per group). All animals were acclimatized for 7 days before the experiment to confirm that there was no abnormality in the animals. Before the operation, we injected lidocaine (1.0 mL/kg) along the auricular vein of rabbits for anesthesia. The rabbits in the KOA group underwent protease (collagenase) injection (1.0 mL/kg, Sigma‐Aldrich, St. Louis, MO) into the articular cavity of their right knees under arthroscopy to induce KOA^15^. The same surgical techniques except for injection of saline instead of collagenase were applied to the rabbits of the control group. The right knee joint of the experimental group was immobilized within 1 week after the injection of collagenase. After the operation, all rabbits were allowed unrestricted activity and food and water and libitum in a pathogen‐free cage. Before injection of collagenase and 30 days after injection, a rabbit was randomly selected from the experimental group and the control group, respectively, for X‐ray and pathological examination of the right knee joint. The protocol was approved by the Animal Care and Use Committee of the Third Xiangya Hospital of Central South University (No 2018‐S233). According to the experimental design, we collected serum and knee joint samples.

### *Radiological Parameters*

The radiological evaluation of knee OA was carried out using X‐rays (Hitachi Limited, DHF‐155HII, Tokyo, Japan). Specimens (rabbits) in the two groups were collected at 4 weeks after the injection of collagenase for radiological evaluation. The images of patients were evaluated using the Kellgren–Lawrence (K‐L) grading scale^16^. The K‐L scoring criteria use a scale from 0 to 4: 0 = normal; 1 = suspicious narrowing of joint space and possible osteophytic lipping; 2 = definite presence of osteophytes, definite narrowing of joint space; 3 = moderate multiple osteophytes, definite narrowing of joint space, some sclerosis, and possible deformity of the bone contour; 4 = large osteophytes, marked narrowing of joint space, severe sclerosis, and clear deformity of the bone contour. The K‐L score of all the radiographs was read by two blinded expert readers.

### *Histological Analysis*

After knee joints were separated, they were soaked and fixed with 10% neutral formalin solution. Then, the specimens of the condyle and cartilage were decalcified with 10% ethylenediamine tetra acetic acid, dehydrated with a series of ethanol washes, and embedded in conventional paraffin. Subsequently, paraffin covered with joint tissue was cut into approximately 6 TTM thick slices (using a microtome [Leica RM2145; Leica, Wetzlar, Germany]) followed by hematoxylin and eosin staining and immunohistochemical staining^17^. Articular cartilage degeneration was evaluated using the modified Mankin scoring system^18^.

### *Serum and Urine Collection*

Serum collection: Serum samples were collected from rabbits in the sham operation and OA groups preoperatively as well as 0, 2, 4, 6, 8, 10, and 12 weeks (6 rabbits in each group) postoperatively^19^. After the blood was centrifuged, the supernatant was collected to evaluate the serum levels of CTX‐II and IL‐1β.

Urine collection: Morning urine (5–10 mL) of all patients and healthy individuals was collected and centrifuged. The supernatant was used to detect the serum concentration of CTX‐II and IL‐1β.

### *Measurement of C‐terminal Telopeptide of Collagen Type II and Interleukin‐1β Concentrations in Urine and Serum*

The quantitative sandwich enzyme immunoassay technique was used to evaluate the serum CTX‐II and IL‐1β concentrations according to the manufacturer guidelines (CTX‐II ELISA Kit and IL‐1β ELISA Kit, Biorbyt, UK). The capture antibody solution (10 g/mL) was added to the plate and incubated overnight. After the standard or sample was added to the plate, the detection antibody dilution and enzyme conjugates were added one after another. The substrate solution was added, followed by stop solution. Subsequently, the optical density (OD) of the plate was read at 450 nm using a Thermo Fisher Scientific Microplate Reader. The CTX‐II and IL‐1β concentrations were calculated by comparing the OD of samples to the standard curve.

### *Statistical Analysis*

The data were expressed as the means ± standard deviation and analyzed using SPSS software, version 22.0 (IBM, USA). Results were analyzed using independent sample *t*‐tests for the K‐L score, the CTX‐II/IL‐1β concentration and the Mankin score, to compare KOA grades/time points with the control group. Spearman's rank correlation analysis was used to evaluate the connection between K‐L score and the CTX‐II and IL‐1β concentration, and between CTX‐II and IL‐1β concentrations. For correlation analysis, 0 < *r*‐value < 1 indicates a positive correlation and −1 < *r*‐value < 0 means a negative correlation between groups. For all determinations, *P*‐values <0.05 were considered statistically significant differences.

## Results

### *General Results of All Subjects and X‐Ray Examination of Arthritis Patients*

#### *General Results of All Subjects*

A summary of the patients and volunteers is presented in Table 1. A total of 25 patients comprising 12 men and 13 women and 9 healthy individuals comprising 4 men and 5 women with a mean age of years (range, 48–69 years) were included. The mean ages of mild, moderate, severe arthritis patients and healthy individuals were 54.29 ± 5.76, 58.44 ± 6.44, 59.89 ± 6.75, and 56.67 ± 4.18 years. The mean body mass index (BMI) of mild, moderate, and severe arthritis patients and healthy individuals were 23.59 ± 1.56, 23.57 ± 2.06, 24.46 ± 1.64, and 23.42 ± 1.35 kg/m^2^. There was no significant difference in the mean ages and BMI among the four groups.

**Table 1:** Demographics of KOA patients and volunteers

Parameters	Values			
	Control	Mild KOA	Moderate KOA	Severe KOA
Gender (male/female)	4/5	3/4	4/5	5/4
Ages (years)	56.67 ± 4.18	54.29 ± 5.76	58.44 ± 6.44	59.89 ± 6.75
Weight (kg)	61.7 ± 5.75	62.41 ± 9.71	63.27 ± 10.94	65.68 ± 8.08
Height (cm)	162.32 ± 8.22	162.16 ± 8.39	163.23 ± 9.27	163.71 ± 9.11
BMI (kg/m^2^)	23.42 ± 1.35	23.59 ± 1.56	23.57 ± 2.06	24.46 ± 1.64

The general results of knee osteoarthritis (KOA) patients and volunteers (control). The gender (male/female), age, weight, height, and body mass index (BMI) of all subjects are summarized in Table 1 (*N* = 9 for control, *N* = 7 for mild arthritis, *N* = 9 for moderate group, *N* = 9 for severe group).

#### *X‐Ray Examination of Arthritis Patients*

**Figure 1:**
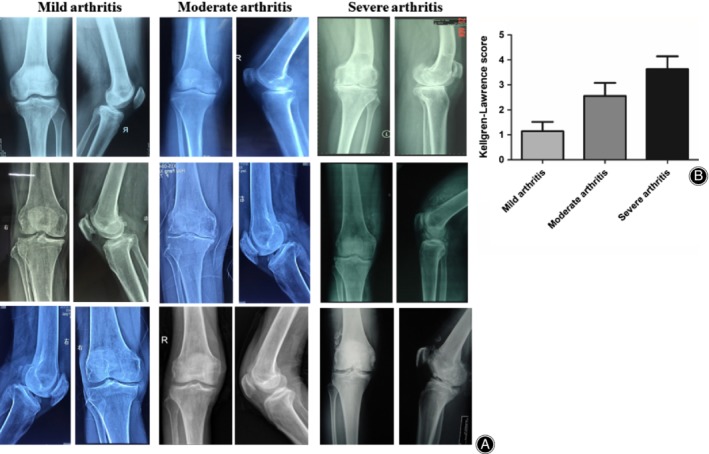
X‐ray examination and the Kellgren–Lawrence (K‐L) scores of arthritis patients. (A) Representative X‐rays from arthritis patients of three degrees. (B) The K‐L scores of three groups. Results are mean values ± SD of samples in three groups (*N* = 7 for mild arthritis, *N* = 9 for moderate group, *N* = 9 for severe group).

The typical images of all patients in three different degrees are showed in Fig. 1A. The K‐L scores of all subjects were evaluated (Fig. 1B). The K‐L score increased gradually with the aggravation of KOA. The K‐L scores of mild, moderate, and severe KOA patients were 1.14 ± 0.38, 2.56 ± 0.53, and 3.63 ± 0.52, respectively.

### *C‐terminal Telopeptide of Collagen Type II and Interleukin‐1β Concentrations in Urine of All Subjects*

**Figure 2:**
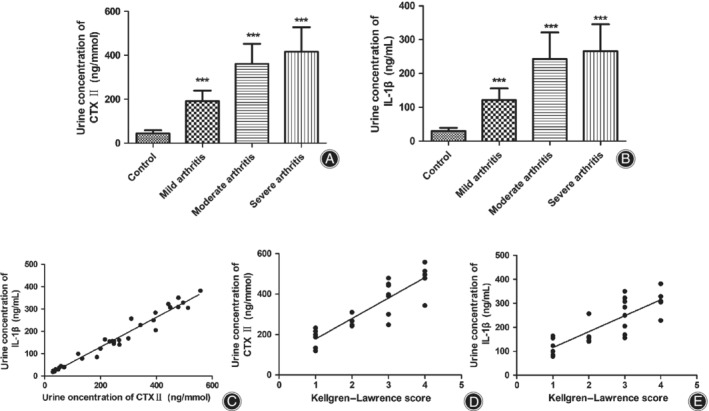
The urine levels of C‐terminal telopeptide of collagen type II (CTX‐II) and interleukin‐1β (IL‐1β) and the correlation analysis. (A, B) Urine level of CTX‐II and IL‐1β in all subjects (***P < 0.001 *vs* control group. *N* = 9 for control group, *N* = 7 for mild arthritis, *N* = 9 for moderate group, *N* = 9 for severe group). (C) The correlation analysis between the urine levels of IL‐1β and CTX‐II of all subjects (*r* = 0.974, *P* < 0.001). (D) The correlation analysis between the Kellgren–Lawrence (K‐L) score and the urine levels of CTX‐II of all patients (*r* = 0.900, *P* < 0.001). (E) The correlation analysis between the K‐L score and the urine levels of IL‐1β of all patients (*r* = 0.813, *P* < 0.001). Data was analyzed with Spearman's rank correlation analysis.

We detected the urine concentration of CTX‐II and IL‐1β in 9 healthy volunteers and all patients. Compared with the control group, the urine concentrations of CTX‐II in patients with KOA in mild, moderate, and severe degrees increased by 3.31, 7.15, and 8.38 times (Fig. 2A, *P* < 0.001). Moreover, the urine concentrations of IL‐1β in mild, moderate, and severe KOA patients were 4.06, 8.12, and 8.90 times higher than that in the control group (Fig. 2B, *P* < 0.001). However, there were no significant difference in urine concentrations of CTX‐II and IL‐1β between moderate and severe groups. The KOA symptoms of patients became more severe, with not only an increased K‐L score, but also elevated concentrations of CTX‐II and IL‐1β. Moreover, there was a positive correlation between the urine concentrations of CTX‐II and IL‐1β (*r* = 0.974, *P* < 0.001, Fig. 2C, KOA patients and volunteers). In addition, the correlation analysis suggested that there was a positive correlation between the urine concentrations of CTX‐II and K‐L score (*r* = 0.900, *P* < 0.001, Fig. 2D, KOA patients). Similarly, there was a positive correlation between the urine concentrations of IL‐1β and K‐L score (*r* = 0.813, *P* < 0.001, Fig. 2E, KOA patients).

### *Identification of Knee Osteoarthritis Rabbit Model*

**Figure 3:**
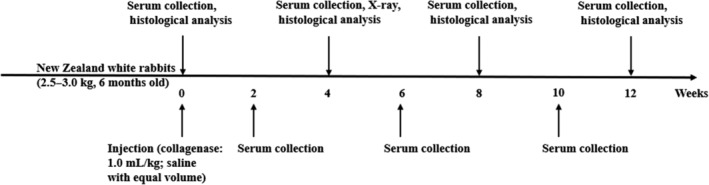
The overview chart of the design in this experimental knee osteoarthritis (KOA) study. Serum samples were collected at different time points. X‐rays were assessed at 4 weeks and histologic analysis was assessed at 0, 4, 8, and 12 weeks.

The overview chart of the design in the experimental KOA is shown in Fig. 3. Results of radiological and pathological examination suggested the successful KOA model in rabbits.

#### *X‐Ray Examination*

**Figure 4:**
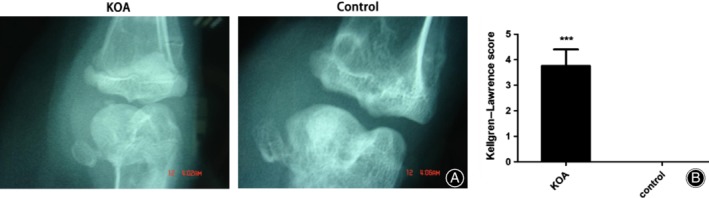
X‐ray examination and the Kellgren–Lawrence (K‐L) scores of the KOA model. (A) Representative X‐rays from two groups. (B) The K‐L scores of both KOA and control groups. Results are mean values ± SD of samples in two groups. (KOA: knee osteoarthritis with collagenase injection; control: saline group) (***P < 0.001 *vs* control group; *N* = 6 for KOA and control group).

X‐ray examination revealed obviously swollen knee joints, narrowing of the joint space, joint marginal hyperplasia, and osteophyte formation accompanied by cystic degeneration (Fig. 4A). The funding is in line with imaging diagnostic criteria for osteoarthritis. The K‐L score in the animals from the KOA group (3.76 ± 0.64) was significantly higher than that of the animals from the control group (0) (Fig. 4B).

#### *Pathological Examination*

**Figure 5:**
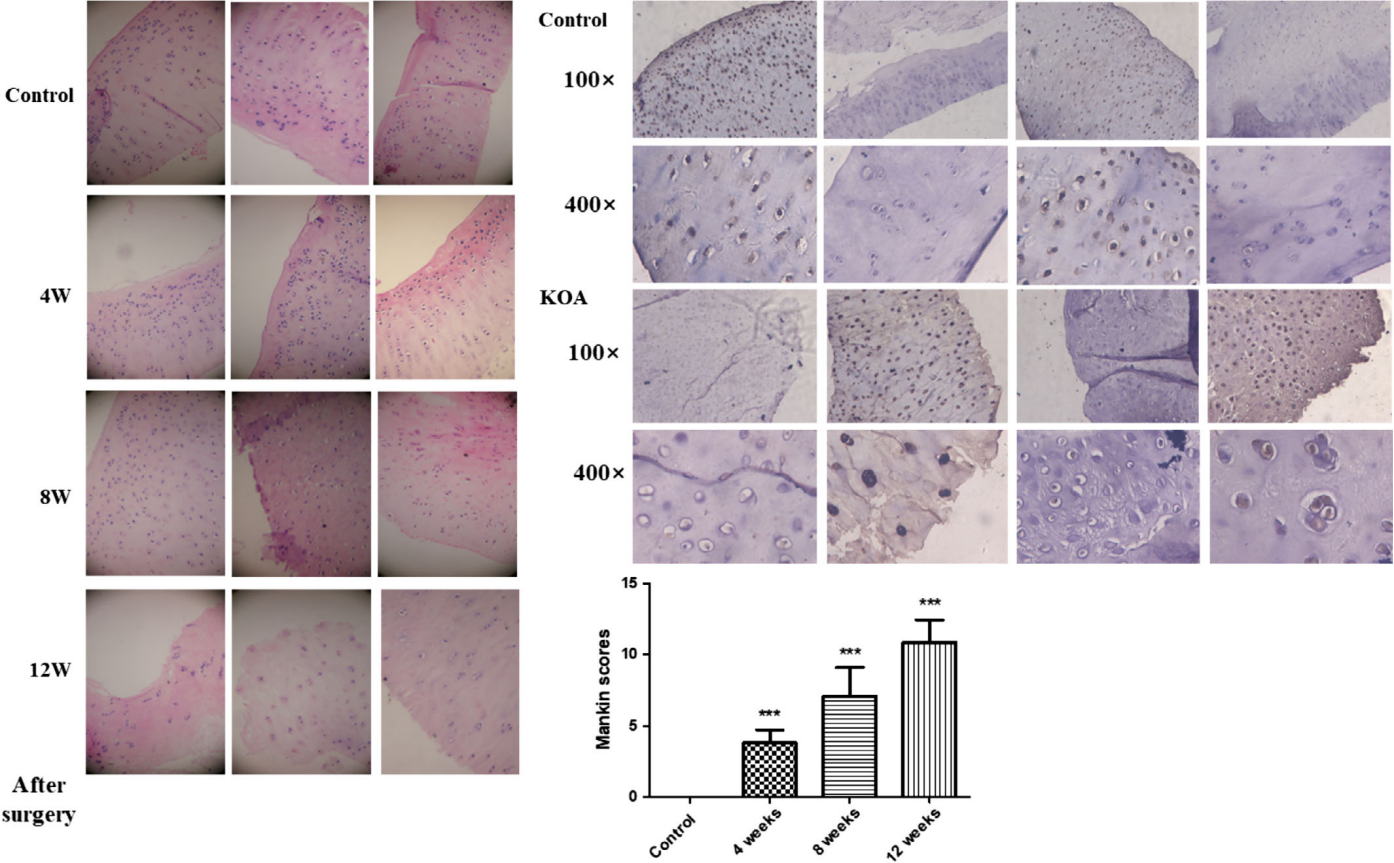
The pathological examination of the KOA model. (A) Typical view (H&E staining) of the articular cartilage from rabbits in two groups (100×). (B) Immunohistochemical images of chondrocyte in two groups (100×, 400×). (C) The Mankin scores of two groups. Results are mean values ± SD of samples in two groups. (KOA: knee osteoarthritis with collagenase injection; control: saline group) (***P < 0.001 *vs* control group; *N* = 6 for KOA and control group).

The data in Fig. 5A showed the typical view (H&E staining) of the articular cartilage from rabbits in two groups (KOA group includes 4, 8, and 12 weeks after surgery). The smooth cartilage surface, columnar cluster of chondrocytes, and complete tidal marking were observed in the slices of HE staining in the control group. However, in the KOA group, the articular cartilage exhibited different manifestations at various stages. At week 4 after surgery, the chondrocytes in the superficial zone were significantly increased and distributed unequally, with a slightly uneven articular cartilage surface and slight distorted tidal marking. Unsmooth articular surface, an irregular tidemark, and disorganized chondrocytes in the middle and deep zone were observed in the 8 weeks postoperatively. At week 12 after surgery, the main characteristics of the cartilage included proliferative chondrocyte clusters in the superficial and middle zone, partial disappearance of tidal marking, and noted cell loss in the middle and deep zone. The typical images (immune‐histochemistry) of articular cartilage in Fig. 5B revealed the smooth cartilage surface, columnar cluster of chondrocytes, and complete tidal marking in the control group, and irregular cartilage surface, disappearing or atrophic chondrocytes, and disappearance of tidal marking in the KOA group. As shown in Fig. 5C, the Mankin scores at each time point in the KOA group were significantly higher than those in the control group. In addition, the Mankin scores of the KOA group were increased during the progression of KOA. The Mankin scores in the control and 4 weeks, 8 weeks, and12 weeks after surgery were 0, 3.79 ± 0.94, 7.06 ± 2.05, and 10.87 ± 1.59. The above results indicated that the KOA model was successful.

### *C‐terminal Telopeptide of Collagen Type II and Interleukin‐1β Concentrations in Serum of Rabbits*

**Figure 6:**
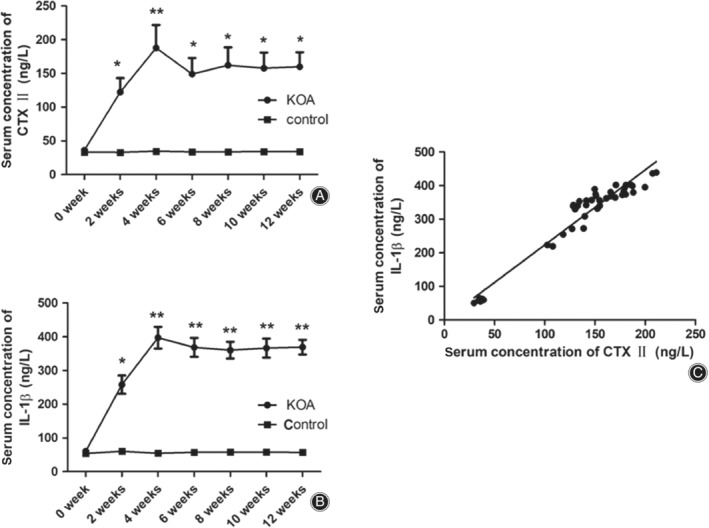
The serum level of C‐terminal telopeptide of collagen type II (CTX‐II) and interleukin‐1β (IL‐1β) and the correlation between them. (A) Serum level of CTX‐II over the course of the experiment for both control and KOA groups. (B) Serum IL‐1β levels at various time points for all animals of two groups. (KOA: knee osteoarthritis with collagenase injection, Control: saline group) (*P < 0. 05, **P < 0.01 *vs* control group; *N* = 6 for KOA and control group) (C) The correlation analysis between the serum levels of IL‐1β and serum CTX‐II. Data was analyzed with Spearman's rank correlation analysis (*r* = 0.967, *P* < 0.001).

The articular fluid was transparent in the control group but turbid and yellowish in the KOA group. The serum concentrations of CTX‐II and IL‐1β in the two groups at various time points are summarized in Fig. 6. The concentration of serum CTX‐II increased from 33.12 ng/L in the control group to 122.36 ng/L in the KOA group, a 3.69‐fold increase at 2 weeks after surgery. The concentration in the KOA group reached a peak (187.78 ng/L) at 4 weeks (Fig. 6A) after surgery. Then, the CTX‐II concentration in the KOA group was slightly reduced until 12 weeks after surgery, which was around 4.5 times that in the control group. Similarly, the serum concentration of IL‐1β in the KOA group was significantly higher than that in the control group except at the preoperational time point (Fig. 6B). The level of IL‐1β in the KOA group was 4.25 times that of the control group at 2 weeks after surgery and 7.23 times at 4 weeks. After that, until 12 weeks after surgery, the IL‐1β concentration in serum of the KOA group remained around 6.3 times that in the control group. In addition, the correlation analysis suggested that there was a positive correlation between the serum concentrations of IL‐1β and serum CTX‐II (*r* = 0.967, *P* < 0.001) (Fig. 6C).

## Discussion

Osteoarthritis, which frequently occurs in the knee joint, is a complex, multifactorial, chronic joint aging disease causing joint pain and disability^20^, ^21^. The main pathological characteristic of OA is the degeneration of cartilage cells and matrix^22^. In the present study, we first found that there was a positive correlation between the K‐L score and the urine concentration of biomolecules markers (CTX‐II and IL‐1β) in clinical KOA patients. Moreover, the data showed that the level of CTX‐II and IL‐1β was significantly increased during the process of KOA, especially in the 4th week after injecting collagenase. This indicated that CTX‐II and IL‐1β can be used as biomolecular markers to provide clues for early diagnosis of KOA.

Type II collagen accounts for 80%–90% of the total collagen in articular cartilage^23^. Type II collagen forms fibrous network with other cartilage collagen, and the destruction of this network is the basis of early OA^24^. CTX‐II is produced by type II collagen through the action of proteases and accumulates during articular cartilage injury of degradation^25^, ^26^, ^27^, ^28^. Many studies have shown that the content of CTX‐II can reflect the degradation degree of articular cartilage. There was a positive correlation between the content of CTX‐II and the degree of cartilage degeneration in OA patients^29^, ^30^. Before the image changed, the level of CTX‐II in OA patients had been significantly higher than that in normal subjects^31^. Studies have shown the correlation between the serum concentration of CTX II and the incidence of KOA, and the risk of progression in early KOA^32^, ^33^. In our study, the CTX‐II concentration in serum and urine, as compared to the control group, was higher in the KOA group. Moreover, there was a positive correlation between the K‐L score and the level of CTX‐II. Our results can be verified with previous studies and reveal its role as an early biomolecular marker in OA.

In addition, we investigated the level of IL‐1β in serum. IL‐1β, a classical inflammatory regulator, can be produced by fibroblasts, chondrocytes, macrophages, synovial cells, and endothelial cells, and plays a central role in OA pathogenesis. There are trace amounts of IL‐1β in the synovial fluid of normal people. However, the level of IL‐1β in synovium and the middle and upper layer chondrocytes and their matrix in cartilage of patients with OA is significantly higher than that of healthy people^34^, ^35^. Normal metabolism and collagen synthesis in the chondrocytes of articular cartilage are disrupted by excessive interleukins (especially IL‐1β and IL‐6), tumor necrosis factor alpha (TNF‐a), and other cytokines which are produced by the increased number of inflammatory cells^6^, ^36^, ^37^, ^38^. Moreover, the IL‐1β production of chondrocytes and synovial cells from knee joints of OA patients *in vitro* is elevated, and there is a positive correlation between the level of IL‐1β in synovial fluid and the degree of articular cartilage injury^39^, ^40^. Our results showed that the level of IL‐1β in the KOA group was significantly increased and there was a positive correlation with the K‐L score. The increase of IL‐1β may be associated with the increase of serum granulocyte/macrophage‐colony stimulating factor (GM‐CSF) in patients with OA^41^. Our results suggested that IL‐1β may partly reflect the early changes of OA.

When OA occurs, the degradation of type II collagen exceeds its synthesis, resulting in the increase of CTX‐II in serum^42^. The fragment of type II collagen induces inflammation, promotes the upregulation of IL‐1β, and leads to apoptosis of chondrocyte^43^. IL‐1β has been shown to induce cartilage matrix degradation, which inhibits proteoglycan and collagen synthesis and promotes the production of matrix metalloproteinase (MMP)^44^, ^45^. Current evidence suggests that the breakdown of extra cartilage matrix is associated with MMP and MMP contribute to the release of CTX‐II^10^, ^46^. Therefore, we speculate that there is a connection between CTX‐II and IL‐1β during the pathological process of KOA. In this study, we found that there was a positive correlation between the concentrations of IL‐1β and CTX‐II. This data partly confirmed our speculation.

In this study, the concentration of CTX‐II and IL‐1β had much higher levels in both the clinical arthritis patients and the experimental KOA rabbit model. The two biomolecules markers were positively correlated with K‐L score, and there was also a positive correlation between their levels. Based on the findings of this study, we concluded that CTX‐II and IL‐1β can be used as biomolecular markers to quantify cartilage degradation and provide clues for early diagnosis and treatment of KOA. Next, the concentrations of two biomolecule markers in serum and articular fluid of patients should be observed in our subsequent paper. Of course, the corresponding signaling pathway of the two bimolecular markers in the pathogenesis of KOA also requires further study. In addition, the application of biomarkers in the treatment of clinical patients is a problem that we will continue to pay attention to in the future. These issues are closely related to the clinical application of molecular markers in the diagnosis and treatment of knee arthritis.
